# Efficacy of consensus interferon in treatment of HbeAg-positive chronic hepatitis B: a multicentre, randomized controlled trial

**DOI:** 10.1186/1743-422X-6-99

**Published:** 2009-07-09

**Authors:** YongLi Zheng, LianSan Zhao, TaiXiang Wu, ShuHua Guo, YaGang Chen, TaoYou Zhou

**Affiliations:** 1 Infectious Disease Centre, West China Hospital, Sichuan University, No. 37, Guo Xue Xiang, Chengdu, Sichuan Province, PR China; 2 Department of Epidemiology, West China Hospital of Sichuan University, Chengdu, Sichuan Province, PR China; 3 Department of Infectious Diseases, The Second Affiliated Hospital of ChongQing Medical University, ChongQing, PR China; 4 Center of Infectious Diseases, The First Affiliated Hospital of Medical School of Zhejiang University, Zhejiang Province, PR China; 5 Department of Infectious Diseases, First People's Hospital of Yibin, No. 65, Wen Xing Street, Yibin, Sichuan Province, PR China

## Abstract

**Background:**

Consensus interferon (CIFN) is a newly developed type I interferon.

**Aims:**

This multicentre, controlled trial was conducted to determine the efficacy of CIFN and to compare it with alpha-1b-interferon (IFN-α1b) in the treatment of patients with hepatitis B e antigen (HBeAg)-positive chronic hepatitis B.

**Methods:**

144 Patients were randomly assigned to receive 9 μg CIFN (CIFN group) or 50 μg INF-α1b (IFN-alpha group) subcutaneously 3 times weekly for 24 weeks, followed by 24 weeks of observation. Efficacy was assessed by normalization of serum alanine transaminase (ALT) levels and the non-detectability of serum hepatitis B virus DNA or HBeAg at the end of treatment and 24 weeks after stopping treatment.

**Results:**

There was no statistically significant difference in the serological, virological and biochemical parameters between CIFN and IFN-α1b groups at the end of the therapy and follow-up period (p > 0.05). Overall, at the end of treatment, 7.0% (5/71) and 35.2% (25/71) of patients in the CIFN group showed a complete or partial response compared with 7.4% (5/68) and 33.8% (23/68) of the IFN-alpha group (p = 0.10). At 24 weeks after stopping treatment, 6.9% (5/72) and 37.5% (27/72) of patients in the CIFN group showed complete response or partial response compared with 7.1% (5/70) and 34.3% (24/70) of the IFN-alpha group (p = 0.10).

**Conclusion:**

These findings suggest that 9 μg CIFN is effective in the treatment of patients with HBeAg-positive chronic hepatitis B. It can gradually induce ALT normalization and HBV DNA clearance and HBeAg loss or HBeAg/HBeAb seroconversion.

## Introduction

Hepatitis B, a serious liver disease caused by the hepatitis B virus, can develop into cirrhosis of the liver or liver cancer[1]. Approximately 2 billion people have been infected with hepatitis B virus and more than 350 million individuals worldwide have chronic liver infections. Each year 1.4 million people die from HBV-related liver disease [2].

There is no specific treatment for chronic HBV infection. Some drugs, including interferon and anti-viral agents [3], can help a number of patients with chronic hepatitis B infection [4]. Interferons were first described by Isaacs & Lindenmann in 1957. Interferon-alpha has been used for the treatment of chronic viral hepatitis for more than 20 years [5]. Many large clinical trials [6-8] and a meta-analysis [9] have proved that interferon is effective and safe in the treatment of hepatitis B.

The effectiveness of interferon for patients with hepatitis B has been widely recognized, but the effect is far from satisfactory [10]. Interferon has the advantage of producing a sustained virologic response after a defined, limited course of treatment [11]. However, it has rather severe side effects, and patients generally are not satisfied with their response to interferon [12]. In-depth studies of interferon have been conducted, and many new types of interferon have been used in practice. Consensus interferon (CIFN) is a new and non-natural type I interferon, which is approved by the Federal Drug Administration for treatment of chronic hepatitis C infection. It is a novel bioengineered "consensus" molecule, composed of the most frequently observed amino acid in each corresponding position in the natural alpha interferons. CIFN shares an 89%, 30% and 60% homology with IFN alpha, IFN beta and IFN omega, respectively [13]. In vitro studies have shown that CIFN has a good affinity with the type-1 interferon receptor, and its antiviral activity is 5 to 20 times higher than traditional interferon [14]. Clinical studies show that CIFN is effective for HCV infection [15,16]. Furthermore, several large multi-centre studies showed that patients with hepatitis C who do not respond to ordinary IFN-alpha therapy may benefit from re-administration of CIFN [17,18]. However, none of the the large clinical trials currently underway with CIFN for chronic hepatitis B have yet been reported.

On the basis of these observations, we conducted a multicentre, randomized controlled trial to compare the efficacy and safety of CIFN vs INF-λ1b therapy in patients with chronic hepatitis B.

## Patients and methods

### Patients

A total of 144 Chinese patients were enrolled in the study. All patients were outpatients from the West China Hospital of Sichuan University, the Second Affiliated Hospital of Chongqing Medical University, and Zhejiang Medical University Hospital. All patients signed informed consent and the Ethics Committee approved the protocol and minor changes in the program.

Inclusion criteria:(1) age 18 to 65 years; (2) presence of HBsAg in serum for at least 6 months, and HBeAg-positive at least once during the screening of PCR HBV-DNA ≥ 10^5 ^copies/ml; (3) in the 35 days before treatment, patients must have had at least one occurrence of an ALT ≥ 2 times the upper limit of normal (ULM, 40 IU/L), but the highest ALT must have been ≤ 10 times ULM, and serum total bilirubin must have been lower than the ULM. Exclusion criteria included prior treatment for CHB with IFN-alpha or another antiviral or immunosuppressive drug; presence of antibodies to hepatitis A, C or D virus, or HIV; decompensated liver disease (more than 7 points by Child-Pugh evaluation or serum bilirubin > 2 times ULM, prothrombin time prolongation > 6 seconds, low serum albumin level, or history of ascites, variceal hemorrhage or hepatic encephalopathy); another cause of chronic liver disease; evidence of hepatocellular cancer or other tumors; history of alcoholism and drug abuse; history of a serious disease of the heart, brain, lung, kidney, thyroid or other important organ, the nervous system history, history of autoimmune disease or history of a blood disorder; allergy history; being pregnant, or breast-feeding if female; organ transplant history; any contraindication to the use of IFN-α; or lack of informed consent [19].

## Methods

The study was a multicentre, randomized controlled trial that followed a detailed protocol. Statisticians calculated the number of samples in advance. Subject designer generated the allocation sequence according to a table of random numbers before recruiting the participants; 3 allocation sequence copies were sealed in opaque envelopes, which were kept confidential. The person who generated the allocation sequence did not recruit the participants. Both participants and results were masked during the treatment and follow-up [20]. Consecutive patients who fulfilled the enrolment criteria were randomly divided into 2 groups to receive either 9 μg CIFN (CIFN group) or 50 μg INF-α1b (IFN-alpha group) subcutaneously 3 times weekly for 24 weeks, followed by 24 weeks of observation.

An analysis of efficacy was performed for all patients who completed the appropriate experimental project in a specified time. Serum complete blood count and biochemical profile, as well as hepatitis serology (HBsAg, anti-HBs, HBeAg, anti-HBe and anti-HBc) was obtained at baseline and then every 4 weeks until the end of treatment, and 8, 16, and 24 weeks after treatment. These tests were conducted in a common centre. HBV-DNA was measured quantitatively by real-time polymerase chain reaction (ULM, HBV DNA < 1,000 copies/mL). Hepatitis serology was done using third-generation ELISA test kits (Equipar, Saronno, Italy).

Virologic response was defined as HBeAg seroconversion, loss of HBeAg and non-detectable HBV-DNA level, and biochemical response as normalization of ALT level. The virologic and biochemical responses between the two groups were assessed at the end of treatment (at 24 weeks) and at 24 weeks after the end of treatment (at 48 weeks). Complete response was defined as HBeAg seroconversion, clearance of HBV DNA and normalization of ALT. Partial response was defined as normalization of ALT and no HBV DNA clearance but a reduction in hepatitis B virus deoxyribonucleic acid (HBV DNA) of ≥ log10 copies/mL or no HBeAg seroconversion. No response was defined as no ALT normalization, a decline of HBV-DNA less than 2 log10 copies/mL and no HBeAg seroconversion or no loss of HbeAg [21].

### Statistical Analyses

Statistical Package for the Social Sciences (version 16.0) was used to conduct all statistical tests. Quantitative variables were expressed as mean and standard deviation. Hepatitis B virus DNA was logarithmically transformed for analysis. Quantitative and qualitative parameters were compared using the Mann-Whitney U test and Pearson chi squared tests or Fisher's exact test, respectively. A p value less than 0.05 was considered statistically significant. All statistical tests were 2-sided.

## Results

One hundred forty-four patients were enrolled in the study and all completed treatment. Female patients comprised 28 of 72 participants in the CIFN group and 23 of 72 participants in the IFN-alpha group. At baseline, the level of HBV-DNA was 1.07*10^8 ^(mean) ± 2.90*10^8 ^(S.D.) in the CIFN group and 0.70*10^8 ^± 1.03*10^8 ^in the IFN-alpha group. ALT level was 194.18 ± 84.00 in the CIFN group and 191.61 ± 89.16 in the IFN-alpha group. Patients between the 2 groups had similar baseline characteristics with respect to age, gender, ALT and HBV DNA levels and Case history (see Table 1). No patients withdrew from the study because of side effects. However, 1 case in the CIFN group and 4 cases in the IFN-alpha group had missing data at the end of treatment. All patients in the CIFN group and 70 of 72 in the IFN-alpha group completed the follow-up.

**Table 1:** Baseline Patient Characteristics

Characteristic	CIFN group (n = 72)	IFN-alpha group (n = 72)
Age, years (mean ± S.D.)	24.8 ± 7.1	27.5 ± 5.4
Female, n (%)	28 (38.9)	23 (31.9)
Case history, years (mean ± S.D.)	6.3 ± 1.1	5.9 ± 1.3
ALT level (mean ± S.D.)	194.18 ± 84.00	191.61 ± 89.16
HBV DNA level (mean ± S.D.)	1.07*10^8 ^± 2.90*10^8^	0.70*10^8 ^± 1.03*10^8^

CIFN, consensus interferon; IFN, interferon; S.D., standard deviation

At the end of treatment, ALT normalization was observed in 38 of 71 (53.5%) patients in the CIFN group and 41 of 68 (60.3%) patients in the IFN-alpha group (p > 0.05). Loss of HBV-DNA was found in 25.4% (18 of 71) of the CIFN group patients and 20.6% (14 of 68) of the IFN-alpha group patients. Rates of HBeAg/anti-HBe seroconversion were 14.1% (10/71) in the CIFN group and 22.1% (15/68) in the IFN-alpha group at the end of therapy (p > 0.05). Loss of HBeAg was evident in 14.1% (10/71) of the CIFN group patients and 10.3% (7/68) of the IFN-alpha group patients (see Table 2).

**Table 2:** Virologic, Biochemical Variables of Patients at 24 Weeks' Treatment

Variable	CIFN group (n = 71)	IFN-alpha group (n = 68)
ALT normalization, n (%)	38 (53.5)	41 (60.3)
Loss of HBV-DNA, n (%)	18 (25.4)	14 (20.6)
HBeAg/anti-HBe seroconversion, n (%)	10 (14.1)	15 (22.1)
Loss of HbeAg, n (%)	10 (14.1)	7 (10.3)
Complete response, n (%)	5 (7.0)	5 (7.4)
Partial response, n (%)	25 (35.2)	23 (33.8)

CIFN, consensus interferon; IFN, interferon

At 24 weeks after stopping treatment, ALT normalization was observed in 38 of 72 (52.8%) patients in the CIFN group and 37 of 70 (52.9%) patients in the IFN-alpha group (p > 0.05). Loss of HBV-DNA was found in 23.6% (17 of 72) of the CIFN group patients and 20.0% (14 of 70) of the IFN-alpha group patients. The rates of HBeAg/anti-HBe seroconversion at the end of the follow-up period were 16.7% (12/72) in the CIFN group and 17.1% (12/70) in the IFN-alpha group (p > 0.05). Loss of HBeAg was observed in 15.3% (11/72) of the CIFN group and 14.3% (10/70) of the IFN-alpha group (see Table 3).

**Table 3:** Virologic, Biochemical Variables of Patients at 24 weeks After Treatment

Variable	CIFN group (n = 72)	IFN-alpha group (n = 70)
ALT normalization, n (%)	38 (52.8)	37 (52.9)
Loss of HBV-DNA, n (%)	17 (23.6)	14 (20.0)
HBeAg/anti-HBe seroconversion, n (%)	12 (16.7)	12 (17.1)
Loss of HbeAg, n (%)	11 (15.3)	10 (14.3)
Complete response, n (%)	5 (6.9)	5 (7.1)
Partial response, n (%)	27 (37.5)	24 (34.3)

CIFN, consensus interferon; IFN, interferon

Complete response was observed in 5 of 71 patients in the CIFN group and 5 of 68 patients in the IFN-alpha group (p > 0.05) at the end of treatment. During the follow-up period, complete response was seen in 5 of 72 patients in the CIFN group and 5 of 70 patients in the IFN-alpha group. The rates of partial response between CIFN group and IFN-alpha group were 35.2% (25/71) and 33.8% (23/68) at the end of treatment. During the follow-up period, partial response was observed in 37.5% (27/72) of the CIFN group and 34.3% (24/70) of the IFN-alpha group.

At the end of the treatment and follow-up periods, none of the patients had loss of HBsAg or appearance of anti-HBs.

## Discussion

There is no specific treatment for hepatitis B, a serious and widely epidemic disease. Interferon has a double mechanism of anti-viral properties and immune regulation for viral hepatitis, therefore, it is widely used in the treatment of hepatitis B virus [22]. CIFN has a certain advantage for hepatitis C infection, yet its efficacy in the treatment of patients with hepatitis B has not been clear. This multicentre, randomized, controlled trial was conducted in Chinese patients positive for HBeAg chronic hepatitis B to determine the efficacy of CIFN in these patients.

The results revealed that CIFN is as effective as INF-α1b for HBeAg-positive chronic hepatitis B. No statistically significant difference existed in the serological, virological and biochemical parameters between the CIFN and IFN-α1b groups at the end of therapy or the follow-up period.

The rates of complete or partial response between the CIFN group and the IFN-alpha group were 42.3% (30/71) and 41.2% (28/68) at the end of treatment, and 44.4% (32/72) and 41.4% (29/70) during the follow-up period, respectively (p > 0.05). A decrease in HBV-DNA was found in 42.3% of patients in the CIFN group compared with 41.2% of patients in the IFN-alpha group at the end of treatment. At the end of follow-up, 44.4% of patients in the CIFN group showed a decrease of HBV-DNA compared with 34.3% of the IFN-alpha group (p > 0.05).

At the end of treatment, loss of HBeAg or HBeAg seroconversion was observed in 32.4% of patients in the IFN-alpha group and 28.2% of patients in the CIFN group. At 24 weeks after stopping treatment, 32.0% patients in the CIFN group had a loss of HBeAg or HBeAg seroconversion in comparison with 31.4% patients in the IFN-alpha group.

Interferon is an important drug against the hepatitis B virus and its efficacy has been widely accepted. Many authors have had similar results with interferon as in our trial [23,24]. The rate of sustained virologic response in our study was between 20% and 50%. Many patients with hepatitis B have benefited from interferon treatment. Moreover, it has obvious advantages to HbeAg seroconversion [25,26]. Our study also confirms that it is effective for chronic hepatitis B virus infection.

Based on the aforementioned data, the dosage of alpha-1b interferon was about 5.6 times higher than CIFN. However, it is difficult to distinguish the difference in efficacy between the two agents. It was also confirmed by statistical analysis. On the one hand, the antiviral activity of the same dosage of CIFN was stronger than that of alpha-1b interferon; on the other hand, the necessary dose of CIFN was lower. Therefore, we assessed the safety of 15 μg CIFN for chronic hepatitis B in another trial. Preliminary results indicate that this dose of CIFN was safe and the efficacy of the high dose was superior to that of the low dose.

According to the literature, CIFN has been used for hepatitis C. Treatment of chronic hepatitis C infection with CIFN in relapsers and non-responders to interferon-based therapy has achieved a certain efficacy [27-29]. Our study suggests that CIFN is effective in retreating patients with chronic hepatitis B infection who failed therapy with interferon alpha.

CIFN has been used for patients with not only hepatitis C but also hepatitis B. The disease burden of hepatitis B is more serious in Asian countries, particularly in China. Therefore, CIFN could be an alternative drug for hepatitis B infection in these areas.

In conclusion, the results of this trial demonstrate that a 24-week course of CIFN therapy is effective for patients with HBeAg-positive chronic hepatitis B. Furthermore, it gradually induces sustained ALT normalization and HBV-DNA clearance and HBeAg loss or HBeAg/HBeAb seroconversion. The therapy was well tolerated by all volunteers who completed the treatment and follow-up periods. We will report its safety in another article in detail. We have observed the best indication and individual characteristics of CIFN. Further study of efficacy and safety will be performed after administration to patients in a large-scale trial, and advantages and disadvantages of CIFN will be compared with existing interferon.

## Competing interests

The authors declare that they have no competing interests.

## Authors' contributions

ZLS, ZYL and WTX were responsible for planning the study, analyzing the results and drafting the manuscript. ZYL wrote the manuscript. GSH, CYG, ZTY and ZYL collected the study material and coordinated the study. All authors have read and approved the manuscript.
